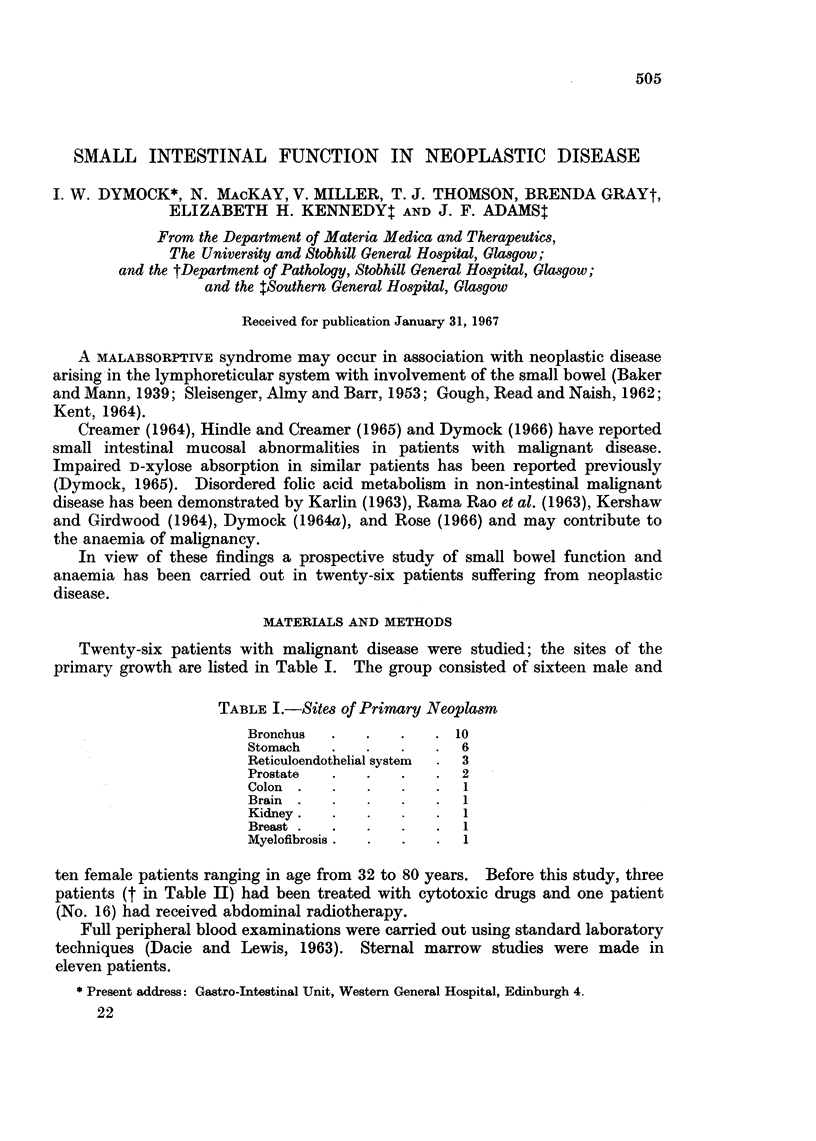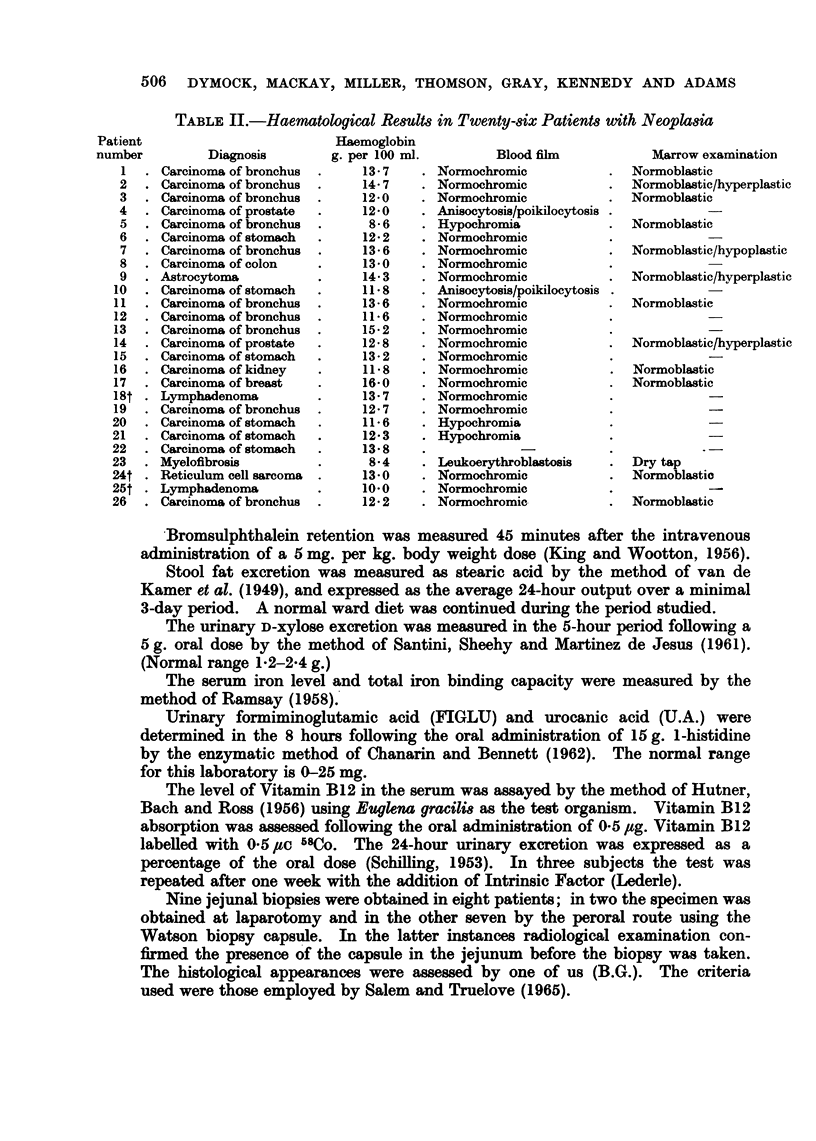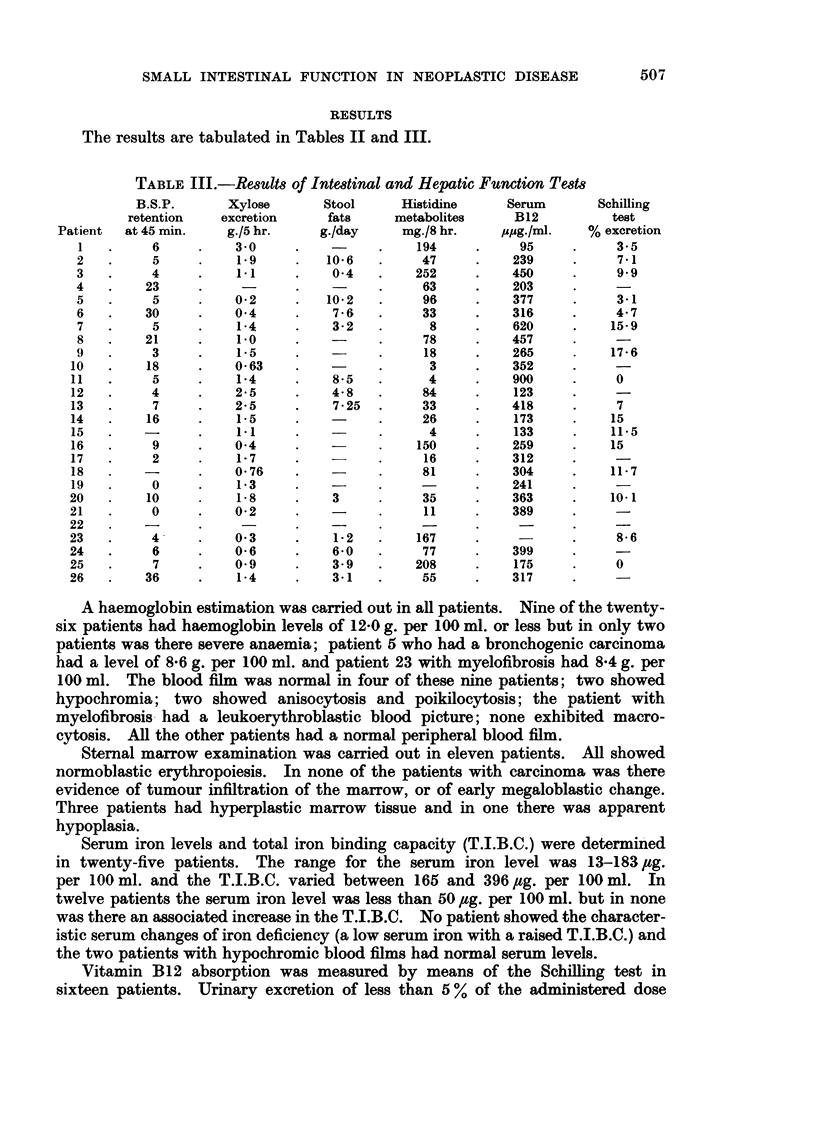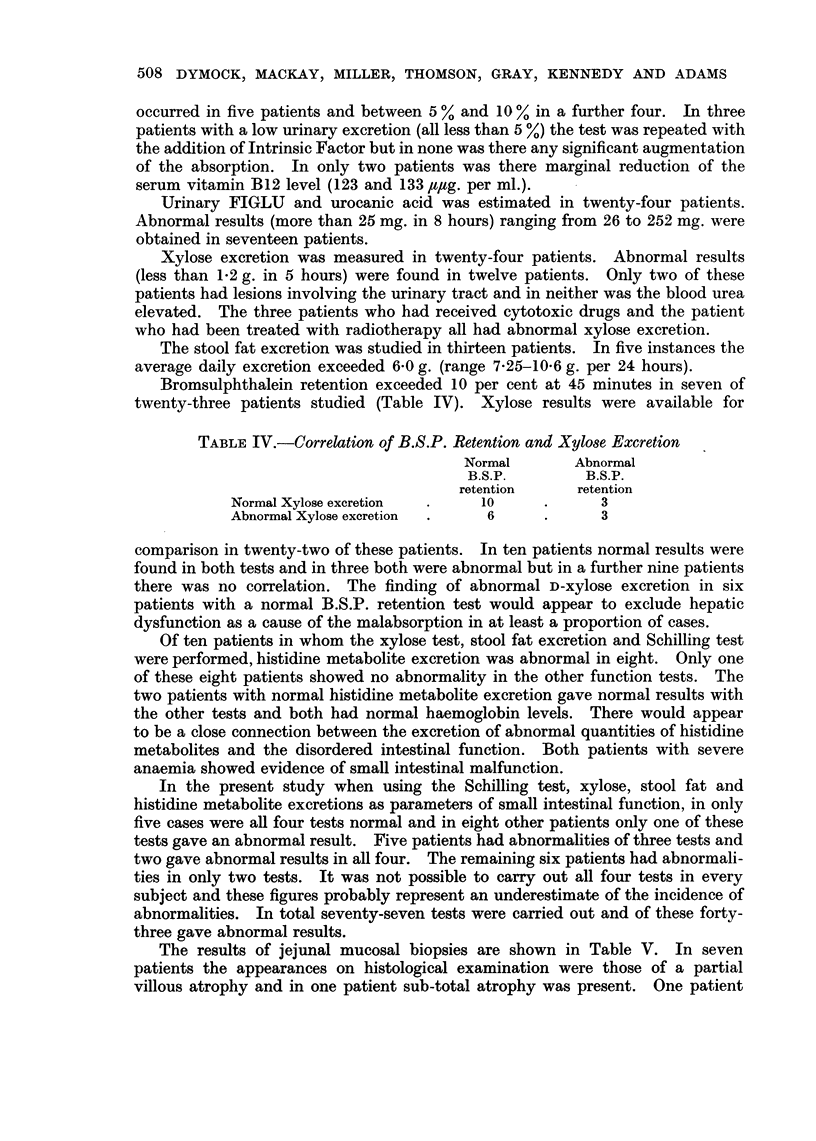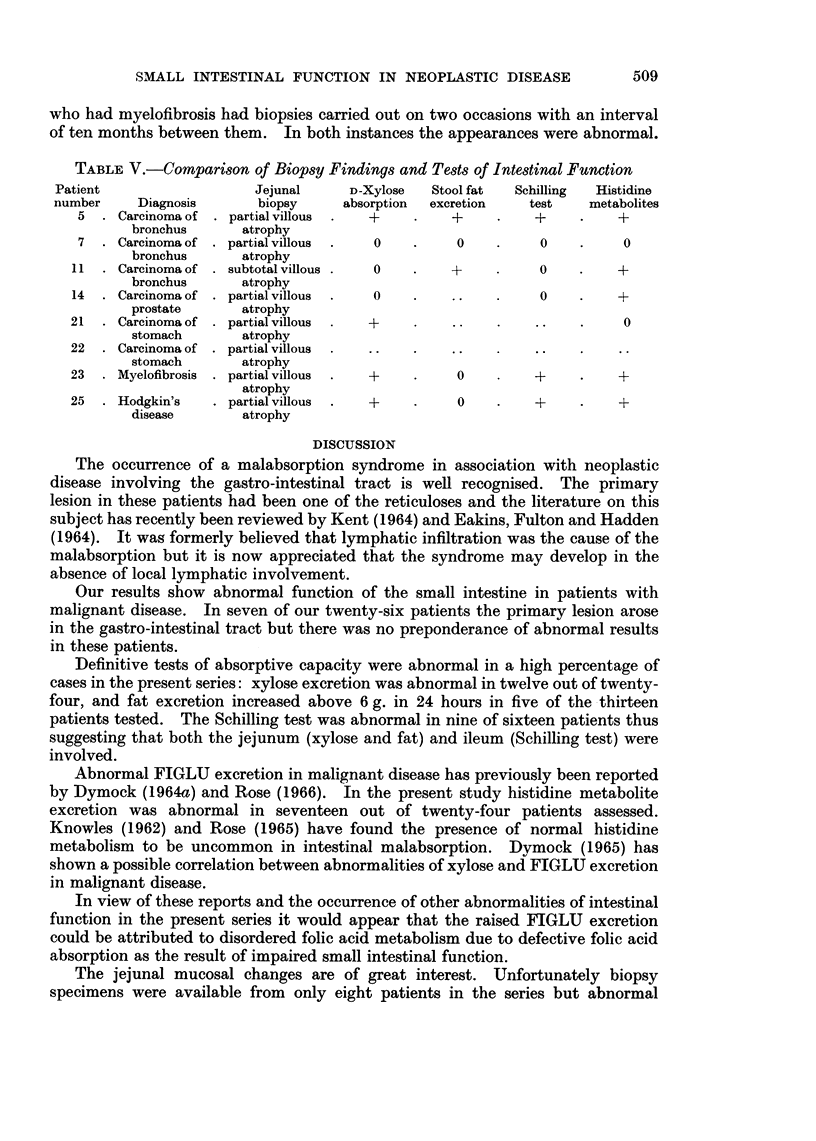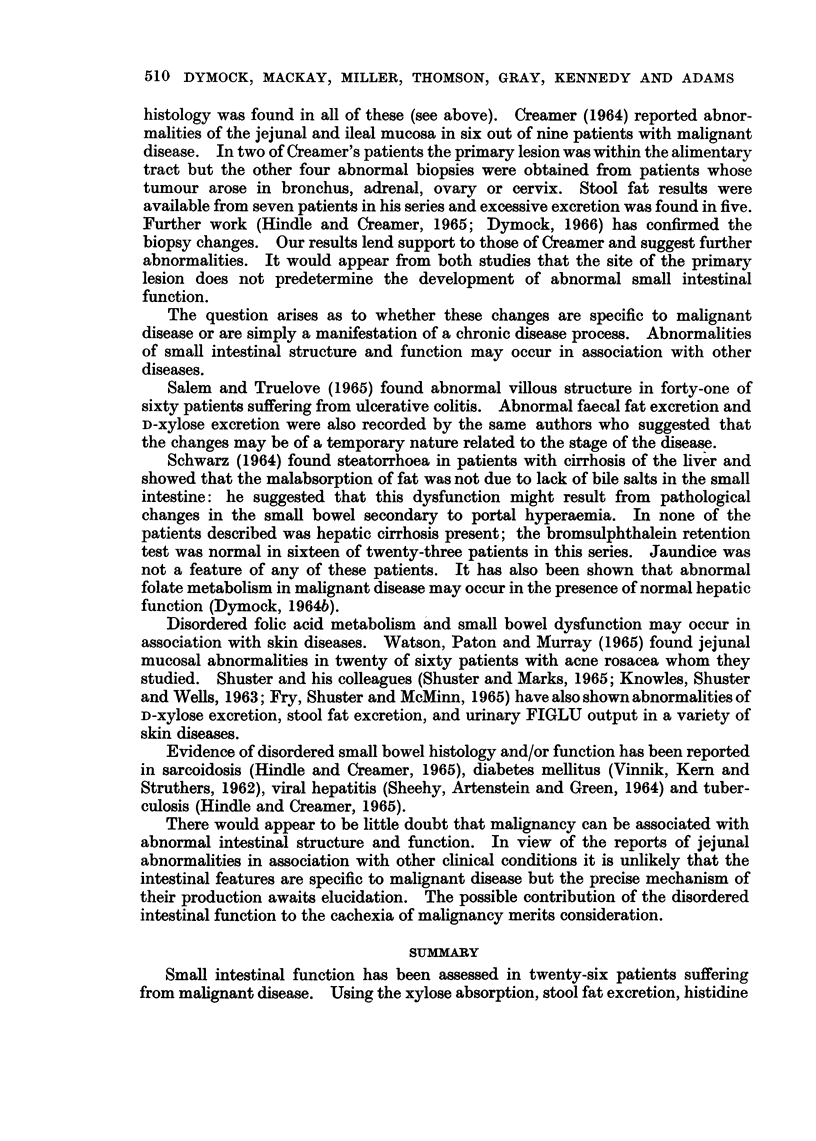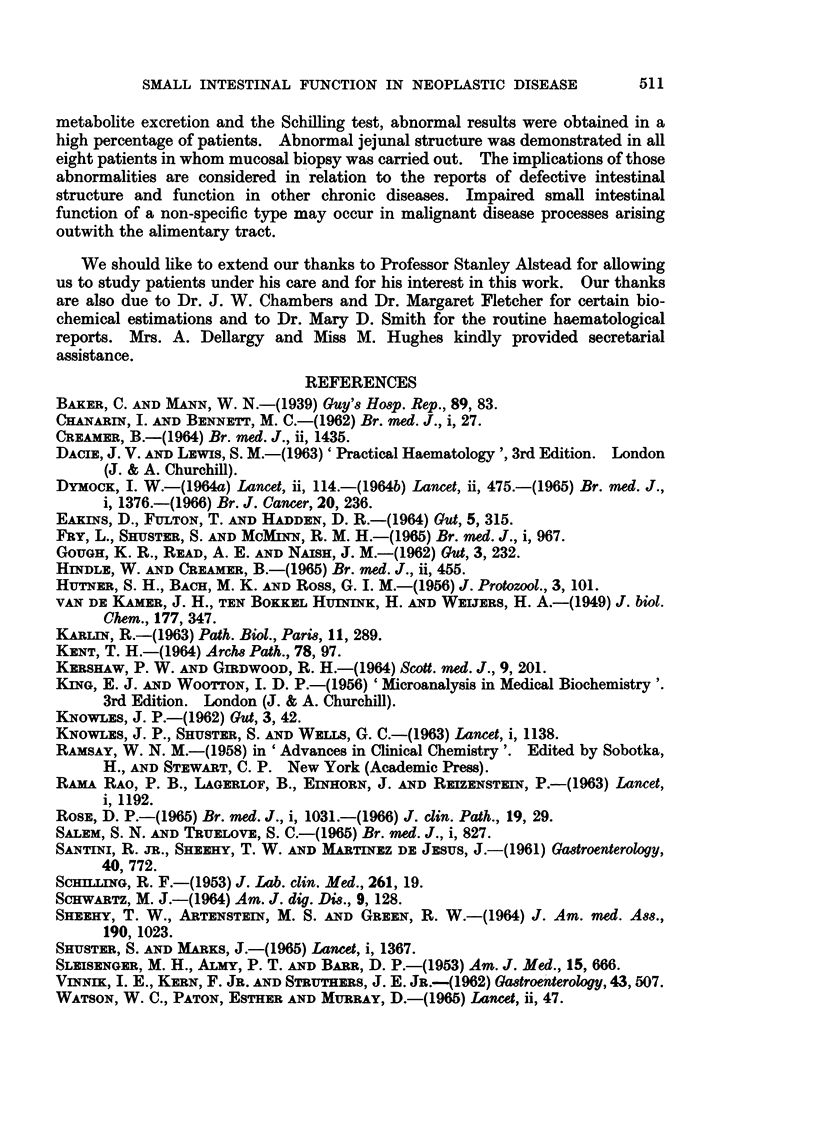# Small intestinal function in neoplastic disease.

**DOI:** 10.1038/bjc.1967.59

**Published:** 1967-09

**Authors:** I. W. Dymock, N. MacKay, V. Miller, T. J. Thomson, B. Gray, E. H. Kennedy, J. F. Adams


### SMALL INTESTINAL FUNCTION IN NEOPLASTIC DISEASE

I. W. DYMOCK*, N. MacKAY, V. MILLER, T. J. THOMSON, BRENDA GRAY†, ELIZABETH H. KENNEDY‡ AND J. F. ADAMS‡

*From the Department of Materia Medica and Therapeutics, The University and Stobhill General Hospital, Glasgow; and the †Department of Pathology, Stobhill General Hospital, Glasgow; and the ‡Southern General Hospital, Glasgow*

Received for publication January 31, 1967

A MALABSORPTIVE syndrome may occur in association with neoplastic disease arising in the lymphoreticular system with involvement of the small bowel (Baker and Mann, 1939; Sleisenger, Almy and Barr, 1953; Gough, Read and Naish, 1962; Kent, 1964).

Creamer (1964), Hindle and Creamer (1965) and Dymock (1966) have reported small intestinal mucosal abnormalities in patients with malignant disease. Impaired D-xylose absorption in similar patients has been reported previously (Dymock, 1965). Disordered folic acid metabolism in non-intestinal malignant disease has been demonstrated by Karlin (1963), Rama Rao *et al.* (1963), Kershaw and Girdwood (1964), Dymock (1964*a*), and Rose (1966) and may contribute to the anaemia of malignancy.

In view of these findings a prospective study of small bowel function and anaemia has been carried out in twenty-six patients suffering from neoplastic disease.

### MATERIALS AND METHODS

Twenty-six patients with malignant disease were studied; the sites of the primary growth are listed in Table I. The group consisted of sixteen male and ten female patients ranging in age from 32 to 80 years. Before this study, three patients († in Table II) had been treated with cytotoxic drugs and one patient (No. 16) had received abdominal radiotherapy.

TABLE I.—*Sites of Primary Neoplasm*

| | |
|---|---|
| Bronchus | 10 |
| Stomach | 6 |
| Reticuloendothelial system | 3 |
| Prostate | 2 |
| Colon | 1 |
| Brain | 1 |
| Kidney | 1 |
| Breast | 1 |
| Myelofibrosis | 1 |

Full peripheral blood examinations were carried out using standard laboratory techniques (Dacie and Lewis, 1963). Sternal marrow studies were made in eleven patients.

* Present address: Gastro-Intestinal Unit, Western General Hospital, Edinburgh 4.

TABLE II.—*Haematological Results in Twenty-six Patients with Neoplasia*

| Patient number | Diagnosis | Haemoglobin g. per 100 ml. | Blood film | Marrow examination |
|---|---|---|---|---|
| 1 | Carcinoma of bronchus | 13·7 | Normochromic | Normoblastic |
| 2 | Carcinoma of bronchus | 14·7 | Normochromic | Normoblastic/hyperplastic |
| 3 | Carcinoma of bronchus | 12·0 | Normochromic | Normoblastic |
| 4 | Carcinoma of prostate | 12·0 | Anisocytosis/poikilocytosis | — |
| 5 | Carcinoma of bronchus | 8·6 | Hypochromia | Normoblastic |
| 6 | Carcinoma of stomach | 12·2 | Normochromic | — |
| 7 | Carcinoma of bronchus | 13·6 | Normochromic | Normoblastic/hypoplastic |
| 8 | Carcinoma of colon | 13·0 | Normochromic | — |
| 9 | Astrocytoma | 14·3 | Normochromic | Normoblastic/hyperplastic |
| 10 | Carcinoma of stomach | 11·8 | Anisocytosis/poikilocytosis | — |
| 11 | Carcinoma of bronchus | 13·6 | Normochromic | Normoblastic |
| 12 | Carcinoma of bronchus | 11·6 | Normochromic | — |
| 13 | Carcinoma of bronchus | 15·2 | Normochromic | — |
| 14 | Carcinoma of prostate | 12·8 | Normochromic | Normoblastic/hyperplastic |
| 15 | Carcinoma of stomach | 13·2 | Normochromic | — |
| 16 | Carcinoma of kidney | 11·8 | Normochromic | Normoblastic |
| 17 | Carcinoma of breast | 16·0 | Normochromic | Normoblastic |
| 18† | Lymphadenoma | 13·7 | Normochromic | — |
| 19 | Carcinoma of bronchus | 12·7 | Normochromic | — |
| 20 | Carcinoma of stomach | 11·6 | Hypochromia | — |
| 21 | Carcinoma of stomach | 12·3 | Hypochromia | — |
| 22 | Carcinoma of stomach | 13·8 | — | — |
| 23 | Myelofibrosis | 8·4 | Leukoerythroblastosis | Dry tap |
| 24† | Reticulum cell sarcoma | 13·0 | Normochromic | Normoblastic |
| 25† | Lymphadenoma | 10·0 | Normochromic | — |
| 26 | Carcinoma of bronchus | 12·2 | Normochromic | Normoblastic |

Bromsulphthalein retention was measured 45 minutes after the intravenous administration of a 5 mg. per kg. body weight dose (King and Wootton, 1956).

Stool fat excretion was measured as stearic acid by the method of van de Kamer *et al.* (1949), and expressed as the average 24-hour output over a minimal 3-day period. A normal ward diet was continued during the period studied.

The urinary D-xylose excretion was measured in the 5-hour period following a 5 g. oral dose by the method of Santini, Sheehy and Martinez de Jesus (1961). (Normal range 1·2–2·4 g.)

The serum iron level and total iron binding capacity were measured by the method of Ramsay (1958).

Urinary formiminoglutamic acid (FIGLU) and urocanic acid (U.A.) were determined in the 8 hours following the oral administration of 15 g. 1-histidine by the enzymatic method of Chanarin and Bennett (1962). The normal range for this laboratory is 0–25 mg.

The level of Vitamin B12 in the serum was assayed by the method of Hutner, Bach and Ross (1956) using *Euglena gracilis* as the test organism. Vitamin B12 absorption was assessed following the oral administration of 0·5 $\mu$g. Vitamin B12 labelled with 0·5 $\mu$c $^{58}$Co. The 24-hour urinary excretion was expressed as a percentage of the oral dose (Schilling, 1953). In three subjects the test was repeated after one week with the addition of Intrinsic Factor (Lederle).

Nine jejunal biopsies were obtained in eight patients; in two the specimen was obtained at laparotomy and in the other seven by the peroral route using the Watson biopsy capsule. In the latter instances radiological examination confirmed the presence of the capsule in the jejunum before the biopsy was taken. The histological appearances were assessed by one of us (B.G.). The criteria used were those employed by Salem and Truelove (1965).

### RESULTS

The results are tabulated in Tables II and III.

TABLE III.—*Results of Intestinal and Hepatic Function Tests*

| Patient | B.S.P. retention at 45 min. | Xylose excretion g./5 hr. | Stool fats g./day | Histidine metabolites mg./8 hr. | Serum B12 $\mu\mu$g./ml. | Schilling test % excretion |
|---|---|---|---|---|---|---|
| 1 | 6 | 3·0 | — | 194 | 95 | 3·5 |
| 2 | 5 | 1·9 | 10·6 | 47 | 239 | 7·1 |
| 3 | 4 | 1·1 | 0·4 | 252 | 450 | 9·9 |
| 4 | 23 | — | — | 63 | 203 | — |
| 5 | 5 | 0·2 | 10·2 | 96 | 377 | 3·1 |
| 6 | 30 | 0·4 | 7·6 | 33 | 316 | 4·7 |
| 7 | 5 | 1·4 | 3·2 | 8 | 620 | 15·9 |
| 8 | 21 | 1·0 | — | 78 | 457 | — |
| 9 | 3 | 1·5 | — | 18 | 265 | 17·6 |
| 10 | 18 | 0·63 | — | 3 | 352 | — |
| 11 | 5 | 1·4 | 8·5 | 4 | 900 | 0 |
| 12 | 4 | 2·5 | 4·8 | 84 | 123 | — |
| 13 | 7 | 2·5 | 7·25 | 33 | 418 | 7 |
| 14 | 16 | 1·5 | — | 26 | 173 | 15 |
| 15 | — | 1·1 | — | 4 | 133 | 11·5 |
| 16 | 9 | 0·4 | — | 150 | 259 | 15 |
| 17 | 2 | 1·7 | — | 16 | 312 | — |
| 18 | — | 0·76 | — | 81 | 304 | 11·7 |
| 19 | 0 | 1·3 | — | — | 241 | — |
| 20 | 10 | 1·8 | 3 | 35 | 363 | 10·1 |
| 21 | 0 | 0·2 | — | 11 | 389 | — |
| 22 | — | — | — | — | — | — |
| 23 | 4 | 0·3 | 1·2 | 167 | — | 8·6 |
| 24 | 6 | 0·6 | 6·0 | 77 | 399 | — |
| 25 | 7 | 0·9 | 3·9 | 208 | 175 | 0 |
| 26 | 36 | 1·4 | 3·1 | 55 | 317 | — |

A haemoglobin estimation was carried out in all patients. Nine of the twenty-six patients had haemoglobin levels of 12·0 g. per 100 ml. or less but in only two patients was there severe anaemia; patient 5 who had a bronchogenic carcinoma had a level of 8·6 g. per 100 ml. and patient 23 with myelofibrosis had 8·4 g. per 100 ml. The blood film was normal in four of these nine patients; two showed hypochromia; two showed anisocytosis and poikilocytosis; the patient with myelofibrosis had a leukoerythroblastic blood picture; none exhibited macrocytosis. All the other patients had a normal peripheral blood film.

Sternal marrow examination was carried out in eleven patients. All showed normoblastic erythropoiesis. In none of the patients with carcinoma was there evidence of tumour infiltration of the marrow, or of early megaloblastic change. Three patients had hyperplastic marrow tissue and in one there was apparent hypoplasia.

Serum iron levels and total iron binding capacity (T.I.B.C.) were determined in twenty-five patients. The range for the serum iron level was 13–183 $\mu$g. per 100 ml. and the T.I.B.C. varied between 165 and 396 $\mu$g. per 100 ml. In twelve patients the serum iron level was less than 50 $\mu$g. per 100 ml. but in none was there an associated increase in the T.I.B.C. No patient showed the characteristic serum changes of iron deficiency (a low serum iron with a raised T.I.B.C.) and the two patients with hypochromic blood films had normal serum levels.

Vitamin B12 absorption was measured by means of the Schilling test in sixteen patients. Urinary excretion of less than 5 % of the administered dose occurred in five patients and between 5 % and 10 % in a further four. In three patients with a low urinary excretion (all less than 5 %) the test was repeated with the addition of Intrinsic Factor but in none was there any significant augmentation of the absorption. In only two patients was there marginal reduction of the serum vitamin B12 level (123 and 133 $\mu\mu$g. per ml.).

Urinary FIGLU and urocanic acid was estimated in twenty-four patients. Abnormal results (more than 25 mg. in 8 hours) ranging from 26 to 252 mg. were obtained in seventeen patients.

Xylose excretion was measured in twenty-four patients. Abnormal results (less than 1·2 g. in 5 hours) were found in twelve patients. Only two of these patients had lesions involving the urinary tract and in neither was the blood urea elevated. The three patients who had received cytotoxic drugs and the patient who had been treated with radiotherapy all had abnormal xylose excretion.

The stool fat excretion was studied in thirteen patients. In five instances the average daily excretion exceeded 6·0 g. (range 7·25–10·6 g. per 24 hours).

Bromsulphthalein retention exceeded 10 per cent at 45 minutes in seven of twenty-three patients studied (Table IV). Xylose results were available for

TABLE IV.—*Correlation of B.S.P. Retention and Xylose Excretion*

| | Normal B.S.P. retention | Abnormal B.S.P. retention |
|---|---|---|
| Normal Xylose excretion | 10 | 3 |
| Abnormal Xylose excretion | 6 | 3 |

comparison in twenty-two of these patients. In ten patients normal results were found in both tests and in three both were abnormal but in a further nine patients there was no correlation. The finding of abnormal D-xylose excretion in six patients with a normal B.S.P. retention test would appear to exclude hepatic dysfunction as a cause of the malabsorption in at least a proportion of cases.

Of ten patients in whom the xylose test, stool fat excretion and Schilling test were performed, histidine metabolite excretion was abnormal in eight. Only one of these eight patients showed no abnormality in the other function tests. The two patients with normal histidine metabolite excretion gave normal results with the other tests and both had normal haemoglobin levels. There would appear to be a close connection between the excretion of abnormal quantities of histidine metabolites and the disordered intestinal function. Both patients with severe anaemia showed evidence of small intestinal malfunction.

In the present study when using the Schilling test, xylose, stool fat and histidine metabolite excretions as parameters of small intestinal function, in only five cases were all four tests normal and in eight other patients only one of these tests gave an abnormal result. Five patients had abnormalities of three tests and two gave abnormal results in all four. The remaining six patients had abnormalities in only two tests. It was not possible to carry out all four tests in every subject and these figures probably represent an underestimate of the incidence of abnormalities. In total seventy-seven tests were carried out and of these forty-three gave abnormal results.

The results of jejunal mucosal biopsies are shown in Table V. In seven patients the appearances on histological examination were those of a partial villous atrophy and in one patient sub-total atrophy was present. One patient who had myelofibrosis had biopsies carried out on two occasions with an interval of ten months between them. In both instances the appearances were abnormal.

TABLE V.—*Comparison of Biopsy Findings and Tests of Intestinal Function*

| Patient number | Diagnosis | Jejunal biopsy | D-Xylose absorption | Stool fat excretion | Schilling test | Histidine metabolites |
|---|---|---|---|---|---|---|
| 5 | Carcinoma of bronchus | partial villous atrophy | + | + | + | + |
| 7 | Carcinoma of bronchus | partial villous atrophy | 0 | 0 | 0 | 0 |
| 11 | Carcinoma of bronchus | subtotal villous atrophy | 0 | + | 0 | + |
| 14 | Carcinoma of prostate | partial villous atrophy | 0 | .. | 0 | + |
| 21 | Carcinoma of stomach | partial villous atrophy | + | .. | .. | 0 |
| 22 | Carcinoma of stomach | partial villous atrophy | .. | .. | .. | .. |
| 23 | Myelofibrosis | partial villous atrophy | + | 0 | + | + |
| 25 | Hodgkin's disease | partial villous atrophy | + | 0 | + | + |

### DISCUSSION

The occurrence of a malabsorption syndrome in association with neoplastic disease involving the gastro-intestinal tract is well recognised. The primary lesion in these patients had been one of the reticuloses and the literature on this subject has recently been reviewed by Kent (1964) and Eakins, Fulton and Hadden (1964). It was formerly believed that lymphatic infiltration was the cause of the malabsorption but it is now appreciated that the syndrome may develop in the absence of local lymphatic involvement.

Our results show abnormal function of the small intestine in patients with malignant disease. In seven of our twenty-six patients the primary lesion arose in the gastro-intestinal tract but there was no preponderance of abnormal results in these patients.

Definitive tests of absorptive capacity were abnormal in a high percentage of cases in the present series: xylose excretion was abnormal in twelve out of twenty-four, and fat excretion increased above 6 g. in 24 hours in five of the thirteen patients tested. The Schilling test was abnormal in nine of sixteen patients thus suggesting that both the jejunum (xylose and fat) and ileum (Schilling test) were involved.

Abnormal FIGLU excretion in malignant disease has previously been reported by Dymock (1964*a*) and Rose (1966). In the present study histidine metabolite excretion was abnormal in seventeen out of twenty-four patients assessed. Knowles (1962) and Rose (1965) have found the presence of normal histidine metabolism to be uncommon in intestinal malabsorption. Dymock (1965) has shown a possible correlation between abnormalities of xylose and FIGLU excretion in malignant disease.

In view of these reports and the occurrence of other abnormalities of intestinal function in the present series it would appear that the raised FIGLU excretion could be attributed to disordered folic acid metabolism due to defective folic acid absorption as the result of impaired small intestinal function.

The jejunal mucosal changes are of great interest. Unfortunately biopsy specimens were available from only eight patients in the series but abnormal histology was found in all of these (see above). Creamer (1964) reported abnormalities of the jejunal and ileal mucosa in six out of nine patients with malignant disease. In two of Creamer's patients the primary lesion was within the alimentary tract but the other four abnormal biopsies were obtained from patients whose tumour arose in bronchus, adrenal, ovary or cervix. Stool fat results were available from seven patients in his series and excessive excretion was found in five. Further work (Hindle and Creamer, 1965; Dymock, 1966) has confirmed the biopsy changes. Our results lend support to those of Creamer and suggest further abnormalities. It would appear from both studies that the site of the primary lesion does not predetermine the development of abnormal small intestinal function.

The question arises as to whether these changes are specific to malignant disease or are simply a manifestation of a chronic disease process. Abnormalities of small intestinal structure and function may occur in association with other diseases.

Salem and Truelove (1965) found abnormal villous structure in forty-one of sixty patients suffering from ulcerative colitis. Abnormal faecal fat excretion and D-xylose excretion were also recorded by the same authors who suggested that the changes may be of a temporary nature related to the stage of the disease.

Schwarz (1964) found steatorrhoea in patients with cirrhosis of the liver and showed that the malabsorption of fat was not due to lack of bile salts in the small intestine: he suggested that this dysfunction might result from pathological changes in the small bowel secondary to portal hyperaemia. In none of the patients described was hepatic cirrhosis present; the bromsulphthalein retention test was normal in sixteen of twenty-three patients in this series. Jaundice was not a feature of any of these patients. It has also been shown that abnormal folate metabolism in malignant disease may occur in the presence of normal hepatic function (Dymock, 1964*b*).

Disordered folic acid metabolism and small bowel dysfunction may occur in association with skin diseases. Watson, Paton and Murray (1965) found jejunal mucosal abnormalities in twenty of sixty patients with acne rosacea whom they studied. Shuster and his colleagues (Shuster and Marks, 1965; Knowles, Shuster and Wells, 1963; Fry, Shuster and McMinn, 1965) have also shown abnormalities of D-xylose excretion, stool fat excretion, and urinary FIGLU output in a variety of skin diseases.

Evidence of disordered small bowel histology and/or function has been reported in sarcoidosis (Hindle and Creamer, 1965), diabetes mellitus (Vinnik, Kern and Struthers, 1962), viral hepatitis (Sheehy, Artenstein and Green, 1964) and tuberculosis (Hindle and Creamer, 1965).

There would appear to be little doubt that malignancy can be associated with abnormal intestinal structure and function. In view of the reports of jejunal abnormalities in association with other clinical conditions it is unlikely that the intestinal features are specific to malignant disease but the precise mechanism of their production awaits elucidation. The possible contribution of the disordered intestinal function to the cachexia of malignancy merits consideration.

### SUMMARY

Small intestinal function has been assessed in twenty-six patients suffering from malignant disease. Using the xylose absorption, stool fat excretion, histidine metabolite excretion and the Schilling test, abnormal results were obtained in a high percentage of patients. Abnormal jejunal structure was demonstrated in all eight patients in whom mucosal biopsy was carried out. The implications of those abnormalities are considered in relation to the reports of defective intestinal structure and function in other chronic diseases. Impaired small intestinal function of a non-specific type may occur in malignant disease processes arising outwith the alimentary tract.

We should like to extend our thanks to Professor Stanley Alstead for allowing us to study patients under his care and for his interest in this work. Our thanks are also due to Dr. J. W. Chambers and Dr. Margaret Fletcher for certain biochemical estimations and to Dr. Mary D. Smith for the routine haematological reports. Mrs. A. Dellargy and Miss M. Hughes kindly provided secretarial assistance.

### REFERENCES

BAKER, C. AND MANN, W. N.—(1939) *Guy's Hosp. Rep.*, **89**, 83.
CHANARIN, I. AND BENNETT, M. C.—(1962) *Br. med. J.*, i, 27.
CREAMER, B.—(1964) *Br. med. J.*, ii, 1435.
DACIE, J. V. AND LEWIS, S. M.—(1963) 'Practical Haematology', 3rd Edition. London (J. & A. Churchill).
DYMOCK, I. W.—(1964*a*) *Lancet*, ii, 114.—(1964*b*) *Lancet*, ii, 475.—(1965) *Br. med. J.*, i, 1376.—(1966) *Br. J. Cancer*, **20**, 236.
EAKINS, D., FULTON, T. AND HADDEN, D. R.—(1964) *Gut*, **5**, 315.
FRY, L., SHUSTER, S. AND MCMINN, R. M. H.—(1965) *Br. med. J.*, i, 967.
GOUGH, K. R., READ, A. E. AND NAISH, J. M.—(1962) *Gut*, **3**, 232.
HINDLE, W. AND CREAMER, B.—(1965) *Br. med. J.*, ii, 455.
HUTNER, S. H., BACH, M. K. AND ROSS, G. I. M.—(1956) *J. Protozool.*, **3**, 101.
VAN DE KAMER, J. H., TEN BOKKEL HUININK, H. AND WEIJERS, H. A.—(1949) *J. biol. Chem.*, **177**, 347.
KARLIN, R.—(1963) *Path. Biol., Paris*, **11**, 289.
KENT, T. H.—(1964) *Archs Path.*, **78**, 97.
KERSHAW, P. W. AND GIRDWOOD, R. H.—(1964) *Scott. med. J.*, **9**, 201.
KING, E. J. AND WOOTTON, I. D. P.—(1956) 'Microanalysis in Medical Biochemistry'. 3rd Edition. London (J. & A. Churchill).
KNOWLES, J. P.—(1962) *Gut*, **3**, 42.
KNOWLES, J. P., SHUSTER, S. AND WELLS, G. C.—(1963) *Lancet*, i, 1138.
RAMSAY, W. N. M.—(1958) in 'Advances in Clinical Chemistry'. Edited by Sobotka, H., AND STEWART, C. P. New York (Academic Press).
RAMA RAO, P. B., LAGERLOF, B., EINHORN, J. AND REIZENSTEIN, P.—(1963) *Lancet*, i, 1192.
ROSE, D. P.—(1965) *Br. med. J.*, i, 1031.—(1966) *J. clin. Path.*, **19**, 29.
SALEM, S. N. AND TRUELOVE, S. C.—(1965) *Br. med. J.*, i, 827.
SANTINI, R. JR., SHEEHY, T. W. AND MARTINEZ DE JESUS, J.—(1961) *Gastroenterology*, **40**, 772.
SCHILLING, R. F.—(1953) *J. Lab. clin. Med.*, **261**, 19.
SCHWARTZ, M. J.—(1964) *Am. J. dig. Dis.*, **9**, 128.
SHEEHY, T. W., ARTENSTEIN, M. S. AND GREEN, R. W.—(1964) *J. Am. med. Ass.*, **190**, 1023.
SHUSTER, S. AND MARKS, J.—(1965) *Lancet*, i, 1367.
SLEISENGER, M. H., ALMY, P. T. AND BARR, D. P.—(1953) *Am. J. Med.*, **15**, 666.
VINNIK, I. E., KERN, F. JR. AND STRUTHERS, J. E. JR.—(1962) *Gastroenterology*, **43**, 507.
WATSON, W. C., PATON, ESTHER AND MURRAY, D.—(1965) *Lancet*, ii, 47.